# Thermotolerant genes essential for survival at a critical high temperature in thermotolerant ethanologenic *Zymomonas mobilis* TISTR 548

**DOI:** 10.1186/s13068-017-0891-0

**Published:** 2017-08-24

**Authors:** Kannikar Charoensuk, Tomoko Sakurada, Amina Tokiyama, Masayuki Murata, Tomoyuki Kosaka, Pornthap Thanonkeo, Mamoru Yamada

**Affiliations:** 1 Division of Product Development and Management Technology, Faculty of Agro-Industrial Technology, Rajamangala University of Technology Tawan-ok, Chanthaburi Campus, Chanthaburi, 22100 Thailand; 2 Life Science, Graduate School of Science and Technology for Innovation, Yamaguchi University, Ube, 755-8505 Japan; 3 Department of Biological Chemistry, Faculty of Agriculture, Yamaguchi University, 1677-1 Yoshida, Yamaguchi, 753-8515 Japan; 4 Research Center for Thermotolerant Microbial Resources, Yamaguchi University, Yamaguchi, 753-8315 Japan; 5 Department of Biotechnology, Faculty of Technology, Khon Kaen University, Khon Kaen, 40002 Thailand

**Keywords:** *Zymomonas mobilis*, Ethanologenic microbe, Transposon mutagenesis, Thermotolerant gene, Ethanol-tolerant

## Abstract

**Background:**

High-temperature fermentation (HTF) technology is expected to reduce the cost of bioconversion of biomass to fuels or chemicals. For stable HTF, the development of a thermotolerant microbe is indispensable. Elucidation of the molecular mechanism of thermotolerance would enable the thermal stability of microbes to be improved.

**Results:**

Thermotolerant genes that are essential for survival at a critical high temperature (CHT) were identified via transposon mutagenesis in ethanologenic, thermotolerant *Zymomonas mobilis* TISTR 548. Surprisingly, no genes for general heat shock proteins except for *degP* were included. Cells with transposon insertion in these genes showed a defect in growth at around 39 °C but grew normally at 30 °C. Of those, more than 60% were found to be sensitive to ethanol at 30 °C, indicating that the mechanism of thermotolerance partially overlaps with that of ethanol tolerance in the organism. Products of these genes were classified into nine categories of metabolism, membrane stabilization, transporter, DNA repair, tRNA modification, protein quality control, translation control, cell division, and transcriptional regulation.

**Conclusions:**

The thermotolerant genes of *Escherichia coli* and *Acetobacter tropicalis* that had been identified can be functionally classified into 9 categories according to the classification of those of *Z. mobilis*, and the ratio of thermotolerant genes to total genomic genes in *Z. mobilis* is nearly the same as that in *E. coli,* though the ratio in *A. tropicalis* is relatively low. There are 7 conserved thermotolerant genes that are shared by these three or two microbes. These findings suggest that *Z. mobilis* possesses molecular mechanisms for its survival at a CHT that are similar to those in *E. coli* and *A. tropicalis*. The mechanisms may mainly contribute to membrane stabilization, protection and repair of damage of macromolecules and maintenance of cellular metabolism at a CHT. Notably, the contribution of heat shock proteins to such survival seems to be very low.

**Electronic supplementary material:**

The online version of this article (doi:10.1186/s13068-017-0891-0) contains supplementary material, which is available to authorized users.

## Background

*Zymomonas mobilis* is an efficient ethanologenic microbe that has been isolated from sugarcane or alcoholic beverages such as African palm wine, and it causes cider sickness and spoiling of beer [1]. The organism bears an anaerobic catabolism via the Entner–Doudoroff pathway [2], which utilizes 1 mol of glucose to yield 2 mol of pyruvate, which is then decarboxylated to acetaldehyde and reduced to ethanol. Due to its strong metabolic activity and low ATP productivity compared to those of the Emden–Meyerhof pathway in the conventional ethanol producer yeast and high-yield ethanol production as a result of the Entner–Doudoroff pathway [1, 3] as well as the fact that the organism is generally regarded as being safe (GRAS) [4], *Z. mobilis* has been focused for its applications to production of useful materials including ethanol as a biofuel, oligosaccharides as food additives, and levan as a medicine [5, 6].

Since the ethanol fermentation process is exothermic [7, 8], ethanologenic microorganisms are exposed to heat stress in addition to other stresses including ethanol [9, 10]. Heat stress has an impact on their growth or viability [11, 12] to prevent fermentation, and the impact is enhanced in the presence of other inhibiting factors, i.e., low pH, high ethanol concentration, and high osmolarity [13–18]. Thus, thermotolerant *Z. mobilis* is thought to be beneficial for the production of useful materials. *Z. mobilis* TISTR 548 is a thermotolerant strain that can grow even at 39 °C [19–21], which is 5–10 °C higher than the optimum temperature for the same genus [22] and the same species [1, 23], and it can efficiently produce ethanol to an extent similar to that of ZM4 [3]. However, information on the molecular mechanism of the thermotolerance of thermotolerant *Z. mobilis* is limited, though some heat shock proteins have been analyzed [24, 25].

Elucidation of the molecular mechanism of microbial survival at a critical high temperature (CHT) may be useful for the development of high-temperature fermentation systems, which have several advantages including reduction in cooling cost, saving of enzyme cost in simultaneous saccharification and fermentation or prevention of contamination of unfavorable microbes [26, 27]. We thus performed transposon mutagenesis of the thermotolerant *Z. mobilis* TISTR 548 to isolate thermosensitive mutants, each of which is defective of one of the so-called thermotolerant genes. The physiological functions of these genes allow us to decipher the molecular mechanism of its survival at a CHT. Moreover, we may be able to understand the general strategy of Gram-negative bacteria to cope with thermal stresses at their individual CHTs by comparison of the mechanism in *Z. mobilis* as α-proteobacteria with those of other bacteria, *Escherichia coli* as γ-proteobacteria and *Acetobacter tropicalis* as α-proteobacteria, that have been investigated [28, 29]. *E. coli* is intrinsically thermotolerant compared to general mesophilic microbes and used for production of useful materials like amino acids, hormones, or vaccines. *Z. mobilis* TISTR548 and *A. tropicalis* are thermotolerant and efficiently produces ethanol and acetic acid, respectively, at relatively high temperatures [19, 29]. Thus, the knowledge of the general strategy might be applicable for relatively thermosensitive mesophilic microbes that have been utilized for production of useful materials in fermentation companies.

## Results

### Isolation of thermosensitive mutants by transposon mutagenesis in thermotolerant *Z. mobilis*

Thermotolerant *Z. mobilis* strain TISTR 548 was subjected to transposon mutagenesis via *E*. *coli* S17-1 harboring pSUP2021Tn*10* as a donor strain for conjugal mating [30]. The growth levels of about 8000 transconjugants obtained were compared on YPD plates at 30 and 39.5 °C, and thermosensitive ones that exhibited no or almost no growth at the high temperature were selected. They were subjected to repeated examination on YPD plates as a second screening and resultantly obtained 123 thermosensitive isolates were further subjected to the final screening in a YPD liquid medium under a static condition at 30 and 39.5 °C. Eventually, 38 isolates that exhibited defective or very weak growth in the liquid culture at the high temperatures were selected as thermosensitive mutants and were used for the following experiments.

The insertion site of Tn*10* in the genome of each mutant was determined by thermal asymmetric interlaced (TAIL)-PCR followed by nucleotide sequencing. The genomic sequences flanking Tn*10* were analyzed by using public databases to identify a disrupted gene. As a result, out of the 38 thermosensitive mutants, only 26 were found to have a Tn*10* insertion in independent genes and 12 were overlapped (Additional file 1: Table S1). This overlapping suggests that the isolation of thermosensitive mutants was nearly saturated. The 26 thermosensitive mutants including 14 representatives showed impaired growth at 39 or 39.5 °C but a similar level of growth to that of the parental strain at 30 °C (Additional file 1: Figure S1).

The gene organization around each Tn*10*-inserted gene might cause a polar effect of the insertion on the transcription of a downstream gene(s) that is intrinsically transcribed by read-through from an upstream promoter(s). Such an organization was found in 12 of the 26 mutants (Additional file 1: Figure S2). The possibility of such polar effects was thus examined by RT-PCR with total RNA that had been prepared from cells grown at 30 and 39.5 °C (Additional file 1: Figure S3). The data suggest that all genes located downstream of the transposon-inserted genes are expressed at the same levels of expression as those in the parental strain. Therefore, it is thought that the thermosensitive phenotype of the 26 thermosensitive mutants is due to the disruption of each gene inserted by Tn*10*, not due to a polar effect on its downstream gene(s). Taken together, 26 independent thermosensitive mutants were obtained and thus 26 thermotolerant genes were identified in thermotolerant *Z. mobilis* TISTR 548.

### Function and classification of thermotolerant genes in thermotolerant *Z. mobilis*

In order to know the physiological functions of thermotolerant genes, database searching was performed. As a result, out of the 26 thermotolerant genes, 24 genes were functionally annotated and classified into 9 categories of general metabolism, membrane stabilization, transporter, DNA repair, tRNA/rRNA modification, protein quality control, translation control, cell division, and transcriptional regulation (Table 1). The remaining 2 genes encode unknown proteins.

**Table 1:** Classification of thermotolerant genes and characterization of their Tn*10*-inserted mutants in *Z. mobilis* TISTR 548

Category	Tn*10*-inserted gene	Function	Protein type^a^	Growth at high temperature compared with that of parental strain^b^			Sensitivity to ethanol^c^		Effect of MgCl_2_^d^
				38 °C	39 °C	39.5 °C	2.0% (v/v)	2.5% (v/v)	
	(WT, TISTR548)			++++	+++++	+++	++++	++++	−
General	ZZ6_0707	Glucose sorbosone dehydrogenase	S	+	+	−	++++	++++	−
Metabolism (Group A)	ZZ6_1376	5,10-methylenetetrahydrofolate reductase	S	++++	++++	+	+++	+++	+++
Membrane	ZZ6_1146	Glucosamine/fructose 6-phosphate aminotransferase	M	+	+	−	++	++	+++
Stabilization (Group B)	ZZ6_0929	Glycosyltransferase group 1	S	+	−	−	++++	+	++++
	ZZ6_0923	Phospholipase D/transphosphatidylase	M	−	−	−	–	−	–
	ZZ6_1551	Squalene hopene cyclase (Shc)	S	−	−	−	+	−	+++
	ZZ6_1046	Tol/Pal system component TolQ	M	+	+	−	++	++	−
	ZZ6_1043	Tol/Pal system component TolB	S	+	+	+	++++	++++	−
	ZZ6_1254	Protein export membrane protein SecD	M	−	−	−	++	+	++
	ZZ6_1477	Preprotein translocase subunit Tim44	M	−	−	−	++++	++++	−
	ZZ6_0158	Autotransporter secretion inner membrane protein TamB	M	+	−	−	++	++	++++
	ZZ6_1210	Competence protein ComEC	M	−	−	−	+	+	+++
	ZZ6_0840	Hypothetical transmembrane protein	M	−	−	−	++++	+++	++
	ZZ6_0541	Hypothetical transmembrane protein	M	++++	+++	+	++	++	++
Transporter (Group C)	ZZ6_1289	Putative Fe^2+^/Mn^2+^ transporter	M	−	−	−	+++	+++	−
DNA repair (Group D)	ZZ6_0616	DNA repair protein RadC	S	++++	+++	+	+++	+++	−
	ZZ6_0934	Exonuclease VII (XseA)	S	−	−	−	+++	+++	−
	ZZ6_0681	DNA repair protein RadA	S	+	+	−	+++	+++	++
tRNA/rRNA modification (Group E)	ZZ6_0023	tRNA/rRNA methyltransferase (SpoU)	S	+++	++	++	++	++	++
Protein quality control (Group F)	ZZ6_1659	Zn-dependent peptidase	S	++++	+++	++	++++	++++	−
	ZZ6_0980	Serin protease DegP	S	−	−	−	−	−	+
Translation control (Group G)	ZZ6_0702	ATP-dependent helicase HrpB	S	−	−	−	+	−	−
Cell division (Group H)	ZZ6_0979	ParA/MinD-like ATPase	S	−	−	−	++	++	−
Transcriptional regulation (Group I)	ZZ6_0019	Trp repressor-binding protein WrbA	S	−	−	−	+++	++	−
Others	ZZ6_0962	Pseudogene	(S)	+	+	−	++++	++++	++
	ZZ6_0861	Hypothetical protein	S	+	+	−	++	++	−

^a^Protein type was described as described in "Methods" sections.”S” and “M” mean soluble protein and membrane protein, respectively

^b^The growth of representative of isolated mutants was compared to that of the parental strain on 3% YPD plates at 38, 39, and 39.5 °C. The number of “+” indicates the degree of cell growth at high temperature compared to that of the parental strain, while “−” indicates no growth

^c^The tolerance of representative of isolated mutants to ethanol was determined by comparison of growth on 3% YPD plates containing 2.0 and 2.5% (v/v) ethanol. The number of “+” indicates the degree of cell growth at 30 °C under the ethanol stress condition compared to that of the parental strain, while “−” indicates no growth

^d^The effect of MgCl_2_ on the growth of representative of isolated mutants was determined by comparison of growth in 3% YPD liquid medium containing 20 mM MgCl_2_ at 39.5 °C. The number of “+” indicates the following degree of cell growth compared to that of the growth in the absence of MgCl_2_: ++, *P* < 0.05; +++, *P* < 0.01; ++++, *P* < 0.001. “−” indicates no significant improvement of growth by the addition of MgCl_2_

Group A consists of two genes related to general metabolism, ZZ6_0707 and ZZ6_1376, that encode glucose sorbosone dehydrogenase and 5, 10-methylenetetrahydrofolate reductase, respectively. The former oxidizes glucose or sorbosone and belongs to a family that possesses a beta-propeller fold. The best characterized in the family is soluble glucose dehydrogenase from *Acinetobacter calcoaceticus*, which oxidizes glucose to glucono-δ-lactone [31]. The latter catalyzes the conversion of 5,10-methylenetetrahydrofolate, which is used for de novo thymidylate biosynthesis, to 5-methyltetrahydrofolate [32], which is used for methionine biosynthesis [32].

Group B is the largest group that consists of 12 genes related to membrane stabilization or membrane formation. Of these, ZZ6_1146 encodes glucosamine/fructose 6-phosphate aminotransferase, which is the first and rate-limiting enzyme in the hexosamine biosynthetic pathway and catalyzes the formation of glucosamine-6-phosphate using glutamine as an ammonia donor. This amino sugar is essential for the formation of a plethora of glycoconjugates for the peptidoglycan macromolecule in prokaryotes [33]. ZZ6_0929 encodes glycosyltransferase group 1, which is involved in biosynthesis of the lipopolysaccharide (LPS) core [34]. This enzyme has two putative conserved domains: one domain covering 94% of the protein is named GT1_mtfB_like. MtfB (mannosyltransferase B) in *E. coli* has been shown to direct growth of the O9-specific polysaccharide chain [35]. The other covering 53% of the protein is named RfaB and is involved in assembly of the lipopolysaccharide core in *E. coli* [36]. ZZ6_0923 encodes phospholipase D/transphosphatidylase possessing the domain of cardiolipin synthase, which catalyzes phosphatidyl group transfer from one phosphatidylglycerol molecule to another to form cardiolipin and glycerol [37]. The *cls* ^−^ for a defective cardiolipin synthase that shows a low level of cardiolipin in phospholipid composition has been reported [38], and the *cls* gene may be related to membrane stabilization. ZZ6_1551 encodes squalene hopene cyclase, which is a key enzyme for hopanoid biosynthesis and cyclizes squalene to hopene [39]. Hopanoids belong to a triterpene series widespread among prokaryotes and play roles in membrane stabilization. Several different hopanoid derivatives are present in *Z. mobilis* [40]. ZZ6_1046 and ZZ6_1043 encode TolQ and TolB, respectively. Both proteins are components of the Tol–Pal (peptidoglycan-associated lipoprotein) system, which is involved in the maintenance of outer membrane stability [41]. Tol proteins are located in the cell envelope and are thought to be involved in the integration of some outer membrane components such as porins and lipopolysaccharides [42]. ZZ6_1254 encodes a protein-export membrane protein, SecD, in the Sec system, and mutations of the gene exhibit pleiotropic defects in protein export in *E. coli* [43]. ZZ6_1477 encodes a preprotein import (inner membrane) translocase subunit, Tim44. In mitochondria, Tim44 is a component to anchor mHsp70 to the TIM23 channel and associates transiently with the TIM23 complex for import of matrix-localized proteins in mitochondria [44]. ZZ6_0158 encodes an autotransporter secretion inner membrane protein, TamB, that forms a complex of the translocation and assembly module with the outer membrane protein, TamA. The complex functions in translocation of autotransporters across the outer membrane [45]. ZZ6_1210 encodes a competence protein, ComEC, that is a DNA transformation transporter (DNA-T) core component (KEGG). Competent cells generally possess a DNA transport complex that is most likely composed of surface-exposed DNA receptors, which facilitate DNA translocation through the cell wall, membrane pores, and motor molecules that power DNA transport [46]. ZZ6_0840 encodes a hypothetical transmembrane protein that possesses a zinc finger domain at its N-terminal portion and a Hid1 superfamily domain at its middle portion as putative conserved protein domains. Hid1 is a high-temperature-induced dauer-forming protein 1 with many putative transmembrane segments in *Caenorhabditis elegans* [47]. ZZ6_0541 encodes a protein bearing an SH3-like domain (COG3807). There are many SH3-like domain-containing proteins [48], but the function of the domain has not been clarified yet except for SH3-like domain-dependent interaction between CheA and CheW [49].

Group C as transporter includes a single gene, ZZ6_1289, that encodes a putative Fe^2+^/Mn^2+^ transporter, which shares 58% identity to Fe^2+^/Mn^2+^ transporter pcl1 in *Acetobacter pasteurianus*.

Group D consists of genes for DNA repair. ZZ6_0616 encodes the DNA repair protein RadC. RadC functions specifically in recombination repair that is associated with a replication fork and is required for growth-medium-dependent repair of DNA double strand breaks in *E. coli* [50]. ZZ6_0934 encodes XseA, a large subunit of exonuclease VII that is implicated in the resection of a nicked mismatched strand in a methyl-directed mismatch repair pathway [51]. ZZ6_0681 encodes the DNA repair protein RadA. In *E. coli*, RadA is involved in recombination and recombination repair and is likely involved in the stabilization or processing of branched DNA molecules or blocked replication forks [52]. *radA* mutants show a modest decrease in survival after UV or X-irradiation exposure [53].

Group E consists of one gene for tRNA/rRNA modification. ZZ6_0023 encodes SpoU, which is a tRNA/rRNA methyltransferase. This enzyme may contribute to stabilization of the structure of tRNA or ribosome [54]. Analysis of the nucleoside modification pattern of tRNA, 16S rRNA, and 23S rRNA in *E. coli* has shown that the modified nucleoside 2′-*O*-methylguanosine, present in a subset of tRNAs at residue 18, is completely absent in the *spoU* mutant [55].

Group F genes are related to protein quality control. ZZ6_1659 encodes a Zn-dependent peptidase (peptidase with a M16 domain) (KEGG). The M16 family of zinc peptidases comprises a pair of homologous domains that form two halves of a ‘‘clam-shell’’ surrounding the active site, and closure of the clam-shell is required for proteolytic activity [56]. ZZ6_0980 encodes the serine protease DegP, and the orthologue gene has been identified as a thermotolerant gene in *E. coli* and *A. tropicalis* [28, 29]. DegP is a chaperone/serine protease located in the periplasm and acts to remove damaged proteins [57, 58].

Group G consists of one gene for translation control. ZZ6_0702 encodes the ATP-dependent helicase HrpB, that acts as an RNA helicase. Some in this helicase group unwind RNA molecules with a 3′ to 5′ polarity [59]. HrpA is an orthologue of HrpB involved in mRNA processing in *E. coli*. *hrpA* mutations in regions for predicted binding and hydrolysis of nucleotide triphosphate abolish the ability for mRNA processing [60].

Group H as cell division includes ZZ6_0979 for ParA/MinD-like ATPase. In *E. coli*, MinD activates a MinC-dependent mechanism responsible for the inactivation of potential division sites and renders the division inhibition system sensitive to MinE, which are required for correct placement of a division site [61]. MinD binds ATP and bears ATPase activity. On the other hand, ParA is required for the equipartition of P1 plasmids during cell division [62].

Group I consists of one gene related to transcriptional regulation. ZZ6_0019 encodes the flavoprotein WrbA, that binds to the tryptophan repressor TrpR and functions as an accessory element in blocking the TrpR-specific transcriptional process [63]. WrbA enhances the formation and/or stabilization of noncovalent complexes between TrpR holorepressor and its primary operator targets [64]. WrbA also functions as an NAD(P)H/quinone oxidoreductase [64] and belongs to the family of multimeric flavodoxin-like proteins [65] as a new type (type IV) of NAD(P)H:quinone oxidoreductase, which protects cells against oxidative stress [64] and may prepare cells for long-term maintenance under stress conditions [66].

There are two genes that deviate from the 9 categories. ZZ6_0962 is named as a pseudogene but should have a crucial function at a high temperature as observed in this study. The pseudogene has an inserted transposon in the gene, but the contribution of the transposon to thermotolerance is unknown. ZZ6_0861 encodes a hypothetical small protein consisting of 82 amino acid residues.

### Effect of supplemented MgCl_2_ on growth of thermosensitive mutants

Mg^2+^ is known to stabilize the outer membrane structure in cells by binding extracellularly [67] and the thermosensitive phenotype of mutants due to the disruption of genes for membrane stabilization is suppressed by the addition of MgCl_2_ at a CHT in *E. coli* [28]. Thus, the effect of MgCl_2_ on growth of thermosensitive mutants in *Z. mobilis* was tested at its CHT.

Thermosensitive mutants and the parental strain were grown in YPD medium with or without 20 mM MgCl_2_ at 39.5 °C for 24 h under a static condition (Additional file 1: Figure S4; Table 1). The growth of 13 thermosensitive mutants was significantly improved by the supplementation of MgCl_2_, 120–260% of that of the parental strain. Eight of them were in Group B and have disrupted genes for membrane stabilization or membrane formation. These results suggest that Mg^2+^ stabilizes the membrane structure at a CHT and protects cells from heat, as has been proposed in *E. coli*.

### Effect of ethanol stress on growth of thermosensitive mutants

*Zymomonas mobilis* as an efficient ethanol producer is often exposed to ethanol stress under fermentation conditions. The effect of exogenous ethanol on thermosensitive mutants was thus examined on YPD plates containing 2.0 or 2.5% ethanol at 30 °C. In consequence, about half of the thermosensitive mutants exhibited repressed growth in the presence of ethanol, less than 50% growth compared to that in the absence of ethanol (Table 1). Interestingly, most of the thermosensitive mutants that were classified into the membrane stabilization group exhibited sensitivity to ethanol stress, and most of the ethanol-sensitive mutants were classified into the group in which the thermosensitive growth phenotype was suppressed by the addition of MgCl_2_. Therefore, these results suggest that the mechanism of thermotolerance at a CHT partially overlaps with that of ethanol stress resistance and allows us to speculate that stabilization of the membrane structure is one of crucial points for ethanol tolerance.

## Discussion

In this study, we isolated 38 thermosensitive mutants by transposon mutagenesis and finally identified 26 thermotolerant genes that are required for survival at a CHT in thermotolerant *Z. mobilis* TISTR 548. Physiological functions and classification of these gene products may allow us to obtain a clue regarding the thermotolerance mechanism of this organism. The gene products were classified into 9 categories (Table 1). About half of them are related to membrane stabilization or membrane formation including enzymes for peptidoglycan or lipid biosynthesis and proteins for protein secretion systems. Most of these, genes for glucosamine/fructose 6-phosphate aminotransferase (ZZ6_1146), glycosyltransferase (ZZ6_0929), squalene hopene cyclase (ZZ6_1551), protein export membrane protein SecD (ZZ6_1254), autotransporter secretion inner membrane protein TamB (ZZ6_0158), competence protein ComEC (ZZ6_1210), hypothetical transmembrane protein (ZZ6_0840), and hypothetical transmembrane protein (ZZ6_0541) were found to be required for ethanol tolerance. Therefore, it is thought that membrane stabilization and maintenance are essential for survival at a CHT. Surprisingly, as found in *E. coli* [28], there was no heat shock protein in these thermotolerant gene products except for DegP, suggesting that not all heat shock proteins may be essential for survival under high temperatures. DegP, which functions in the periplasm as a chaperone at low temperatures and as a protease at high temperatures [68], is thought to play a role in the maintenance of homeostasis of the periplasm or membranes. In *E. coli*, *groEL* as an essential gene was induced at a CHT [28] and thus some heat shock proteins may be required under such an extreme condition.

Thermotolerant genes have also been identified in *E. coli* BW25113 and *A. tropicalis* SKU1100: 72 and 24 genes, respectively [28, 29; unpublished data]. The thermotolerant genes of the two microbes can be classified into 9 categories according to the classification of those of *Z. mobilis*, and the number and distribution of these genes are shown in Table 2. The ratios of thermotolerant genes to total genomic genes in *Z. mobilis*, *E. coli*, and *A. tropicalis* are 1.47, 1.68, and 0.70%, respectively. We do not know the reason why the ratio in *A. tropicalis* is relatively low. In the case of *E. coli*, a single-gene knockout library was used for screening thermosensitive mutants and thus almost all of the genes except for essential genes were examined. On the other hand, in the case of *Z. mobilis* and *A. tropicalis*, transposon mutagenesis was applied for screening thermosensitive mutants, and the ratios of the number of thermotolerant genes, for each of which two or more transposon-inserted mutants were isolated, to the total number of thermotolerant genes (Additional file 1: Table S1) [29] were 35 and 21%, respectively. Therefore, the low ratio of multiple mutants for the same gene in *A. tropicalis* suggests the possibility that there are still unidentified thermotolerant genes in *A. tropicalis* SKU1100. In all categories except for general metabolism, ratios of thermotolerant genes in *Z. mobilis* are closer to those in *E. coli* than those in *A. tropicalis*. Notably, *Z. mobilis* has a higher ratio of thermotolerant genes for membrane stabilization than the ratios in other two microbes: 46, 25, and 20% in *Z. mobilis*, *E. coli*, and *A. tropicalis*, respectively.

**Table 2:** Comparison of thermotolerant genes among *Z. mobilis* TISTR 548, *E. coli* BW25113, and *A. tropicalis* SKU1100

Category	No. of thermotolerant gene (ratio %^a^)		
	*Z. mobilis*	*E. coli* ^b^	*A. tropicalis* ^c^
General metabolism	2 (0.11%)	22 (0.51%)	1 (0.03%)
Membrane stabilization	12 (0.68%)	18 (0.42%)	5 (0.15%)
Transporter	1 (0.06%)	3 (0.07%)	3 (0.09%)
DNA repair and DNA modification	3 (0.17%)	6 (0.14%)	1 (0.03%)
tRNA and rRNA modification	1 (0.06%)	9 (0.21%)	0 (0%)
Protein quality control and stress response	2 (0.11%)	4 (0.09%)	5 (0.15%)
Translational control	1 (0.06%)	3 (0.07%)	2 (0.06%)
Cell division	1 (0.06%)	3 (0.07%)	2 (0.06%)
Transcriptional regulation	1 (0.06%)	0 (0%)	2 (0.06%)
Others	2 (0.11%)	3 (0.07%)	4 (0.12%)
Sum of thermotolerant gene	26 (1.47%)	72 (1.68%)	24 (0.70%)
Total genomic genes	1765	4288	3412

^a^Ratio was estimated using the number of total genomic genes

^b^Data of Murata et al. [28] and unpublished data

^c^Data of Soemphol et al. [29]

On the other hand, *E. coli* possesses several discriminating sets of thermotolerant genes, which are absent in the other two microbes: 4 genes (*aceE*, *aceF*, *lpd*, and *lipA*) for pyruvate metabolism, 3 genes (*atpA*, *atpD*, and *atpG*) for ATPase, 3 genes (*cydB*, *yhcB*, and *cydD*) for ubiquinol oxidase or its formation, and 3 genes (*ubiE*, *ubiH*, and *ubiX*) for ubiquinone biosynthesis in the category of general metabolism, 8 genes (*gmhB*, *lpcA*, *rfaC*, *rfaD*, *afaE*, *rfaF*, *rfaG*, and *lpxL*) for lipopolysaccharide biosynthesis and 5 genes (*ydcL*, *yfdL*, *ynbE*, *nlpI*, and *ycdO*) for peptidoglycan-associated lipoproteins or predicted lipoproteins in the category of membrane stability, 5 genes (*dnaQ*, *holC*, *priA*, *ruvA*, and *ruvC*) for DNA double-strand break repair in the category of DNA repair, and 6 genes (*iscS*, *yheL*, *yheM*, *yheN*, *yhhP*, and *yccM*) for a sulfur relay system in the category of tRNA modification [28; unpublished data]. Of these sets, genes for the lipopolysaccharide biosynthesis and the sulfur relay system are postulated to have been acquired by horizontal gene transfer [28]. The genes in the 4 categories described above seem to contribute to specific strategies for thermotolerance in *E. coli* [28; some thermotolerant genes will be described elsewhere].

There are common thermotolerant genes or thermotolerant genes related to the same physiological function or pathway among the three microbes. In the category of protein quality control, the three microbes share *degP* and both *Z. mobilis* and *A. tropicalis* have a gene for Zn-dependent protease (ZZ6_1659 and ATPR_0429, respectively). In membrane stabilization, one gene related to hopanoid biosynthesis is present in *Z. mobilis* and *A. tropicalis* (*shc* and ATPR_1188, respectively) and two to three genes for the Tol-Pal system are present in *Z. mobilis* (*tolQ* and *tolB*) and *E. coli* (*pal*, *tolQ* and *tolR*). One gene related to MinC-dependent cell division inhibition in cell division is present in *Z. mobilis* and *A. tropicalis* (*minD* and *minC*, respectively), and *wrbA* in transcriptional regulation and *nhaA* for the Na^+^/H^+^ antiporter in transporters are shared by *Z. mobilis* and *A. tropicalis.* On the basis of the functions of these genes and combinations of other thermotolerant genes in each category, some common strategies for thermotolerance have emerged: in the category of membrane stabilization, synthesis or modification of peptidoglycan and maintenance of integrity for all three microbes, and hopanoid or lipid synthesis for *Z. mobilis* and *A. tropicalis*; in DNA repair, double-strand DNA repair, which may be accumulated at a CHT, for *Z. mobilis* and *E. coli*; tRNA modification, probably for a stable structure at such a high temperature, for *Z. mobilis* and *E. coli*; in chaperone and protease, removal of damaged proteins, especially by periplasmic serine protease DegP, for all three microbes; control of chromosome segregation for *E. coli* and *A. tropicalis*, and control of cell division for all three microbes; and in transcriptional regulation, Trp repressor-binding protein WrbA (still unclear why necessary) for *Z. mobilis* and *A. tropicalis.* In addition, import or export of some metal ions may be important probably for keeping homeostasis of some ions, export of toxic ions or maintenance of membrane potential.

At a CHT, several problems including protein unfolding or increase in membrane fluidity occur. Reactive oxygen species increase as the temperature increases [69], causing the damage of macromolecules including DNA [70, 71]. The requirement of genes for the 9 categories allows us to make speculations about various types of damage of membrane and proteins or about the abnormal structures of macromolecules including proteins, DNAs and RNAs at a CHT. Microbes would have thus acquired thermotolerant genes to overcome these problems. Moreover, it is assumed that these genes are involved in the response of cells to other stresses including osmotic stress or oxygen stress. In fact, *Z. mobilis* increases thermotolerance by the addition of sorbitol [72] and exhibits faster growth and higher ethanol production under a static condition than that under a shaking condition [19, unpublished]. Further experiments are required for clarifying this assumption.

## Conclusions

The thermotolerant genes of thermotolerant ethanologenic *Z. mobilis* TISTR 548 have been identified. Comparison with thermotolerant genes in *E. coli* and *A. tropicalis* reveal that these genes of the three microbes can be classified into 9 categories and that there are common thermotolerant genes or thermotolerant genes related to the same physiological function or pathway among the three microbes, which suggest several common strategies, including membrane stabilization, protection and repair of macromolecules of proteins, DNAs and RNAs, and maintenance of cellular metabolism-like cell division, transcription or translation, for the three microbes to survive at CHT. Considering the genetic conversion of non-thermotolerant to thermotolerant bacteria, such strategies might be applicable.

## Methods

### Materials

A DNA sequencing Kit (ABI PRISM ^®^ Terminator v 3.1 Cycle sequencing Kit) was obtained from Applied Biosystem Japan. Oligonucleotide primers were synthesized by Proligo Japan K.K. (Tokyo, Japan). Other chemicals were all of analytical grade and obtained from commercial sources.

### Microorganisms and media

*Zymomonas mobilis* TISTR 548 [19, 20] and its derivatives were grown in YPD (3% glucose, 0.5% peptone, and 0.3% yeast extract) medium. *E. coli* S17-1 harboring pSUP2021 Tn*10* [30] was grown in LB (0.5% yeast extract, 1% NaCl, and 1% Bactotryptone) medium supplemented with 12.5 µg/ml of tetracycline.

### Conjugation and transposon mutagenesis

*Escherichia coli* S17-1 harboring pSUP2021 Tn*10* as a donor for conjugal mating was grown in LB medium containing 12.5 µg/ml of tetracycline under a shaking condition at 100 rpm at 37 °C. The recipient *Z. mobilis* TISTR 548 was grown in YPD medium under a static condition at 30 °C. Cells of both strains were grown to the mid-log phase, washed three times with LB medium, recovered by centrifugation at 5000 rpm for 1 min, and suspended in a small volume of LB medium. Both cell suspensions were then mixed at a ratio of donor and recipient of 3:2 and stood for 3 h at 30 °C. The suspensions were spotted on the surfaces of LB agar plates and incubated at 30 °C for 5 h. After the mating steps, cells were recovered, resuspended in a small volume of YPD medium, and spread on YPD agar plates containing 0.15% acetic acid and 12.5 µg/ml of tetracycline. Transconjugants (transposon-inserted mutants) that appeared on the plates after 3-day incubation at 30 °C were subjected to the following screening.

### Screening of thermosensitive mutants

About 8000 transconjugants were subjected to the first screening in which they were grown at 30 and 39.5 °C on YPD agar plates. Transposon-inserted mutants that showed no or almost no growth on the plates at 39.5 °C were selected for the next screening. The second screening was performed under the same condition as that in the first screening. Selected mutants were then subjected to the last screening in which their thermosensitivity was examined in 2-ml liquid culture of YPD medium at 30 and 39.5 °C for 24 h under a static condition. Cell growth was determined by measuring cell turbidity at OD_550_. Mutants that showed a value at OD_550_ significantly less than that of the parent strain were selected and defined as thermosensitive mutants.

### Examination of the effects of heat and ethanol stresses on growth of thermosensitive mutants

Thermosensitive mutants and the parental strain were pre-cultured in YPD medium under a static condition at 30 °C until a mid-log phase. For the heat stress experiment, the pre-cultured cells were serially diluted with YPD medium, spotted on YPD agar plates, and incubated at 30, 38, 39, and 39.5 °C for 40 h. For the ethanol stress experiment, the pre-cultured cells were serially diluted, spotted on YPD plates supplemented with 2.0 or 2.5% ethanol, and incubated at 30 °C for 40 h. Growth ability was examined in triplicate.

### Effect of Mg^2+^ on growth of thermosensitive mutants

Thermosensitive mutants and the parental strain were pre-cultured in YPD medium under a static condition at 30 °C until a mid-log phase. The pre-cultured cells were inoculated in YPD medium with or without 20 mM MgCl_2_ and incubated at 39.5 °C for 24 h under a static condition. The experiments were performed more than 3 times. The significance of the effect of MgCl_2_ on cell growth was evaluated by a *t* test.

### Identification of the transposon (Tn*10*)-inserted site in a thermosensitive mutant genome by TAIL-PCR followed by nucleotide sequencing

The Tn*10*-inserted site in the genome of each thermosensitive mutant was determined by TAIL-PCR [73] followed by nucleotide sequencing. The genomic DNA from thermosensitive mutants was isolated as described previously [74]. The concentration of isolated genomic DNA was measured by using Nanodrop (Nanodrop Technologies, Wilmington, DE). TAIL-PCR was performed by using TaKaRa PCR Thermal Cycler Dice^®^ mini (TaKaRa). Three specific primers for TAIL-PCR were TnISR-1 (GATCCTCTCGTTTGTTGCGGTCAGGCC) [30], TnISR-1.5 (AGGGCTGCTAAAGGAAGCGG) (this work) and TnISR-2 (ACGAAGCGCAAAGAGGAAGCAGG) [29], and an arbitrary degenerated primer was AD2 (GTNCGASWCANAWGTT) [73]. The first PCR was carried out in a 50-µl mixture containing 10 ng of chromosome DNA, 5.0 µM TnISR-1, 25 µM AD2 primer, 500 µM each of dNTPs, 0.5 U PrimeSTAR (TaKaRa) and 1× buffer supplied for the enzyme. Two percent of the first PCR product was used as a template for the second PCR, which was performed using the same reaction mixture as that used for the first PCR except that TnISR-1.5 was used as a specific primer. The third PCR was also performed using the same reaction mixture as that used for the first PCR except that TnISR2 was used as a specific primer and the concentration of AD2 was reduced to 12.5 µM [25]. The second or third PCR product was purified by using a PCR product purification kit (Qiagen) and subjected to nucleotide sequencing on an ABI PRISM 310 Genetic Analyzer (Applied Biosystems) or DNA Sequencer GenomeLab GeXP (Beckman Coulter). The sequencing reaction was performed with a BigDye^®^ Terminator v3.1 Cycle Sequencing Kit (Applied Biosystems) or a GenomeLab Dye Terminator Cycle Sequencing with Quick Start Kit (Beckman Coulter).

### RT-PCR

*Zymomonas mobilis* cells were grown in 50 ml of YPD medium under a static condition at 30 °C until exponential phase, and then the temperature was increased to 39.5 °C and the cultivation was continued for 8 min. As a control, the cultivation was continued for 8 min at 30 °C. Total RNA was prepared from these heat-stressed or not heat-stressed cells by the hot phenol method [75]. RT-PCR analysis was performed using an mRNA-selective RT-PCR kit (TaKaRa) and primers (Additional file 1: Table S2) to examine the expression of immediate downstream genes of Tn*10*-inserted genes as described previously [28]. The reverse transcription reaction was carried out at 42 °C for 15 min, followed by PCR at 85 °C for 1 min, 45 °C for 1 min, and extension at 72 °C for 1 min, using the two specific primers for each gene. After the completion of 15, 20, 25, and 30 cycles, the PCR products were analyzed by 0.9% agarose gel electrophoresis and stained with ethidium bromide [76]. The relative amounts of RT-PCR products on the gel were compared by measuring the density of bands on the gel by using image J (https://imagej.nih.gov/ij/). Under our conditions, the RNA-selective RT-PCR was able to specifically detect mRNA because no band was observed when reverse transcriptase was omitted.

### Bioinformatics analysis

The intrinsic gene that was inserted by Tn*10* in each thermotolerant mutant was confirmed to be a thermotolerant gene after analyses of the gene organization and/or expression of its downstream gene. Thermotolerant genes were then subjected to functional classification by bioinformatics analysis mainly according to the instructions of KEGG (http://www.genome.jp/kegg/), NCBI (http://www.ncbi.nlm.nih.gov/), Inter Pro (http://www.ebi.ac.uk/interpro/), and Uniprot (http://www.uniprot.org/). Protein type was analyzed by TMHMM (http://www.cbs.dtu.dk/services/TMHMM/). Homology searching and alignment were performed using BLAST [77]. The *Z. mobilis* TISTR 548 thermotolerant genes were designed as ZZ6_XXXX according to *Z*. *mobilis* subsp. *mobilis* ATCC29191 because the genome sequence of TISTR 548 was found to be almost identical to that of ATCC29191 after draft sequencing (unpublished).

## Additional file

**Additional file 1.** Additional figures and tables.

## Abbreviations

HTF: high-temperature fermentation
TISTR: Thailand Institute of Scientific and Technological Research
GRAS: generally regarded as being safe
CHT: critical high temperature
TAIL-PCR: thermal asymmetric interlaced PCR
LPS: lipopolysaccharide
DNA-T: DNA transformation transporter
NADH: reduced form of nicotinamide adenine dinucleotide
NADPH: reduced form of nicotinamide adenine dinucleotide phosphate
TnISR: transposon-inserted region
AD: arbitrary degenerate
