# Supplementary material for: Thermotolerant genes essential for survival at a critical high temperature in thermotolerant ethanologenic Zymomonas mobilis TISTR 548
Source: Biotechnol Biofuels. 2017 Aug 24;10:204. doi: 10.1186/s13068-017-0891-0 (PMC5571576; doi:10.1186/s13068-017-0891-0)
Supplement: Supplementary file 1 — Additional file 1. Additional figures and tables. [file 13068_2017_891_MOESM1_ESM.docx]

Supplementary Information

Thermotolerant genes essential for survival at a critical high temperature in thermotolerant ethanologenic *Zymomonas mobilis* TISTR 548

**Authors:** Kannikar Charoensuk^1^, Tomoko Sakurada^2^, Amina Tokiyama^3^, Masayuki Murata^2^, Tomoyuki Kosaka^2, 3, 4^, Pornthap Thanonkeo^5^ and Mamoru Yamada^2, 3, 4 §^

**Affiliation**

^1^Division of Product Development and Management Technology, Faculty of Agro-Industrial Technology, Rajamangala University of Technology Tawan-ok, Chanthaburi Campus, Chanthaburi 22100, Thailand

^2^Life Science, Graduate School of Science and Technology for Innovation, Yamaguchi University, Ube 755-8505, Japan

^3^Department of Biological Chemistry, Faculty of Agriculture, Yamaguchi University, Yamaguchi 753-8515, Japan

^4^Research Center for Thermotolerant Microbial Resources, Yamaguchi University, Yamaguchi 753-8315, Japan

^5^Department of Biotechnology, Faculty of Technology, Khon Kaen University,

Khon Kaen 40002, Thailand

^§^ **Correspondence to:**

^§^Corresponding author: Mamoru Yamada

Mailing address: Department of Biological Chemistry, Faculty of Agriculture, Yamaguchi University, 1677-1 Yoshida, Yamaguchi 753-8515, Japan. Tel.: +81-83-933-5869; Fax: +81-83-933-5820; e-mail: [m-yamada@yamaguchi-u.ac.jp](mailto:m-yamada@yamaguchi-u.ac.jp)

**Figure S1** Growth comparison among isolated thermosensitive mutants and parental strain.

Squares and circles show the growth of isolated mutants and parental strain, respectively. Each strain was incubated at 30°C, 39°C and 39.5°C under a static condition. At the times indicated, cell density was determined by measuring cell turbidity at OD_550_.

**Figure S2** Gene organization around transposon-inserted genes.

The direction of arrow-boxes shows the direction of transcription.

**Figure S3** Testing of the possibility of polar effects by transposon insertion.

The preparation of total RNA from cells cultured at 30°C (a) and 39.5°C (b) and the procedure of RT-PCR were described in Materials and Methods. RT-PCR was performed with primers specific for a gene just downstream from each thermotolerant gene to amplify about 500-bp DNA fragments. After RT reaction, PCR was performed for 15, 20, 25 and 30 cycles and each PCR product was electrophoresed on 1.2% agarose gel, followed by staining with ethidium bromide. Arrows indicate amplified products by RT-PCR.

**Figure S4** Effect of supplemented MgCl_2_ on growth of thermosensitive mutants.

Mutants and parental strain were grown in 2 ml of 3% YPD medium with or without 20 mM MgCl_2_ at 39.5°C for 24 h under a static condition. The cell density at 24 h was determined by measuring a turbidity at OD_550_. The number of + indicates a significant difference as compared to growth of mutant without MgCl_2_. ++ indicates P < 0.05, +++ indicates P < 0.01, ++++ indicates P < 0.001.

**Table S1 Isolated Tn10-inserted thermosensitive mutants**

Tn10-inserted gene No. of isolated Representative

gene mutant mutant

ZZ6_0707 1 TC01

ZZ6_1376 2 TC03

ZZ6_1146 1 TE12

ZZ6_0929 2 3-24

ZZ6_0923 2 TC04

ZZ6_1551 3 C12-36

ZZ6_1046 2 C11-44

ZZ6_1043 2 1-2

ZZ6_1254 1 C31-23

ZZ6_1477 1 TC15

ZZ6_0158 1 TC14

ZZ6_1210 1 C13-36

ZZ6-0840 1 TC11

ZZ6_0541 1 TC09

ZZ6_1289 1 1-10

ZZ6_0616 1 CF32

ZZ6_0934 2 C12-43

ZZ6_0681 1 TC10

ZZ6_0023 1 C12-44

ZZ6_1659 1 C21-17

ZZ6_0980 4 TC05

ZZ6_0702 1 TC12

ZZ6_0979 1 TE19

ZZ6_0019 2 C31-15

ZZ6_0962 2 C12-37

ZZ6_0861 1 TC13

**Table S2 RT-PCR primers used in this study**

Mutants Primers Sequences

TC01 ZZ6_0706-5’ 5’-ACCGGAATAGATAGCCAGAA-3’

ZZ6_0706-3’ 5’-CTGGTCACAAACATGGAAAC-3’

TC03 ZZ6_1377-5’ 5’-GTTATTGATGCCATTACCCG-3’

ZZ6_1377-3’ 3’-AAAGATTGCGTCCTGACATA-3’

3-24 ZZ6_0930-5’ 5’-TTCTCGATAATGCGACCATAGC-3’

ZZ6_0930-3’ 5’- TGGCAACTTTAAAATCGAACACAA-3’

C11-44 ZZ6_1045-5’ 5’-GTTGATGTGATGTTGGTTCTG-3’

ZZ6_1045-3’ 3’-TGAGCAGCCGATTTATCTG-3’

C31-23 ZZ6_1255-5’ 5’-CCTTGTTGGGTGTAAAAGGA-3’

ZZ6_1255-3’ 5’-TTGTAGCAGCTATTCTGACG-3’

TC15 ZZ6_1478-5’ 5’-CTGTTCATTGCTGTAAACGC-3’

ZZ6_1478-3’ 5’-CTAATTTGGCTGGTTTTGAGC-3’

C13-36 ZZ6_1209-5’ 5’-CGCTCAAGCACAGTTAAATC-3’

ZZ6_1209-3’ 5’-CGTCACCGTTCATAAAAACA-3’

TC11 ZZ6_0841-5’ 5’-ATTTTATGCTCAATATCTTGTGGT-3’

ZZ6_0841-3’ 5’-ACACATCACCATTGGTAAAAC-3’

CF32 ZZ6_0617-5’ 5’-TCGCTACTTGTGCTATTTCA-3’

ZZ6_0617-3’ 5’TGGTTTATCCTGAACGGATG-3’

TC10 ZZ6_0680-5’ 5’-GACCAATTTTCATCCAGTCG-3’

ZZ6_0680-3’ 5’-GAATACCGGCATAATCGAGA-3’

C12-37 ZZ6_0963-5’ 5’-ACCAACAACACTCCCAATAG-3’

ZZ6_0963-3’ 5’-AAAAATATATGGCGATCCTACTG-3’

TC13 ZZ6_0860-5’ 5’-ATGCTGCGTTTACCGATT-3’

ZZ6_0860-3’ 5’-ATTTTGCCATTGGGTACCAT-3’
